# Associations between Genetically Predicted Circulating Protein Concentrations and Endometrial Cancer Risk

**DOI:** 10.3390/cancers13092088

**Published:** 2021-04-26

**Authors:** Jingjing Zhu, Tracy A. O’Mara, Duo Liu, Veronica Wendy Setiawan, Dylan Glubb, Amanda B. Spurdle, Peter A. Fasching, Diether Lambrechts, Daniel Buchanan, Pik Fang Kho, Linda S. Cook, Christine Friedenreich, James V. Lacey, Chu Chen, Nicolas Wentzensen, Immaculata De Vivo, Yan Sun, Jirong Long, Mengmeng Du, Xiao-Ou Shu, Wei Zheng, Lang Wu, Herbert Yu

**Affiliations:** 1 Population Sciences in the Pacific Program, Cancer Epidemiology Division, University of Hawaii Cancer Center, University of Hawaii at Manoa, Honolulu, HI 96813, USA; liuduo95@126.com (D.L.); Lwu@cc.hawaii.edu (L.W.); hyu@cc.hawaii.edu (H.Y.); 2 Department of Genetics and Computational Biology, QIMR Berghofer Medical Research Institute, Brisbane, QLD 4006, Australia; Tracy.OMara@qimrberghofer.edu.au (T.A.O.); Dylan.Glubb@qimrberghofer.edu.au (D.G.); Amanda.Spurdle@qimrberghofer.edu.au (A.B.S.); PikFang.Kho@qimrberghofer.edu.au (P.F.K.); 3 Department of Pharmacy, Harbin Medical University Cancer Hospital, Harbin 150086, China; 4 Department of Preventive Medicine, Keck School of Medicine and Norris Comprehensive Cancer Center, University of Southern California, Los Angeles, CA 90089, USA; vsetiawa@usc.edu; 5 Department of Gynecology and Obstetrics, Comprehensive Cancer Center ER-EMN, University Hospital Erlangen, Friedrich-Alexander-University Erlangen-Nuremberg, 91054 Erlangen, Germany; peter.fasching@fau.de; 6 Department of Medicine Division of Hematology and Oncology, David Geffen School of Medicine, University of California, Los Angeles, CA 90095, USA; 7 Laboratory for Translational Genetics, Department of Human Genetics, University of Leuven, 3000 Leuven, Belgium; diether.lambrechts@kuleuven.vib.be; 8 VIB, VIB Center for Cancer Biology, 3000 Leuven, Belgium; 9 Department of Clinical Pathology, The University of Melbourne, Melbourne, VIC 3010, Australia; daniel.buchanan@unimelb.edu.au; 10 Centre for Epidemiology and Biostatistics, Melbourne School of Population and Global Health, The University of Melbourne, Melbourne, VIC 3010, Australia; 11 Genomic Medicine and Family Cancer Clinic, Royal Melbourne Hospital, Parkville, VIC 3052, Australia; 12 Victorian Comprehensive Cancer Centre, University of Melbourne Centre for Cancer Research, Parkville, VIC 3000, Australia; 13 Epidemiology, Biostatistics and Preventive Medicine, Department of Internal Medicine, University of New Mexico, Albuquerque, NM 87131, USA; lcook@salud.unm.edu; 14 Department of Cancer Epidemiology and Prevention Research, Alberta Health Services, Calgary, AB T2S 3C3, Canada; Christine.Friedenreich@albertahealthservices.ca; 15 Department of Computational and Quantitative Medicine, City of Hope, Duarte, CA 91010, USA; jlacey@coh.org; 16 Epidemiology Program, Fred Hutchinson Cancer Research Center, Seattle, WA 98109, USA; cchen@fhcrc.org; 17 Division of Cancer Epidemiology and Genetics, National Cancer Institute, Bethesda, MD 20892, USA; wentzenn@mail.nih.gov; 18 Department of Epidemiology, Harvard T.H. Chan School of Public Health, Boston, MA 02115, USA; devivo@channing.harvard.edu; 19 Department of Medicine, Harvard Medical School, Channing Division of Network Medicine, Brigham and Women’s Hospital, Boston, MA 02115, USA; 20 Vanderbilt Epidemiology Center, Vanderbilt-Ingram Cancer Center, Department of Medicine, Division of Epidemiology, Vanderbilt University School of Medicine, Nashville, TN 37232, USA; yan.sun@vanderbilt.edu (Y.S.); jirong.long@vumc.org (J.L.); xiao-ou.shu@Vanderbilt.Edu (X.-O.S.); wei.zheng@vumc.org (W.Z.); 21 Department of Epidemiology and Biostatistics, Memorial Sloan Kettering Cancer Center, New York, NY 10065, USA; dumeng@mskcc.org

**Keywords:** genetic instrument, protein biomarker, endometrial cancer, risk

## Abstract

**Simple Summary:**

Endometrial cancer is the leading female reproductive tract cancer in developed countries. Discovering new biomarkers is critical for understanding the etiology this cancer and identifying women with a higher risk of this cancer from the general population. Several blood protein biomarkers have been linked to endometrial cancer in previous studies, but these studies have assessed only a limited number of biomarkers usually among a small number of participants. The current study aimed at identifying novel circulating protein biomarkers of endometrial cancer by using the largest available dataset to date. Our finding suggested nine proteins to be associated with endometrial cancer risk, and five of the identified associations showed suggestive associations with risk of non-endometrioid EC, a much more lethal subtype. If validated by additional studies, our findings may contribute to understanding the pathogenesis of endometrial tumor development and facilitating the risk assessment of endometrial cancer.

**Abstract:**

Endometrial cancer (EC) is the leading female reproductive tract malignancy in developed countries. Currently, genome-wide association studies (GWAS) have identified 17 risk loci for EC. To identify novel EC-associated proteins, we used previously reported protein quantitative trait loci for 1434 plasma proteins as instruments to evaluate associations between genetically predicted circulating protein concentrations and EC risk. We studied 12,906 cases and 108,979 controls of European descent included in the Endometrial Cancer Association Consortium, the Epidemiology of Endometrial Cancer Consortium, and the UK Biobank. We observed associations between genetically predicted concentrations of nine proteins and EC risk at a false discovery rate of <0.05 (*p*-values range from 1.14 × 10^−10^ to 3.04 × 10^−4^). Except for vascular cell adhesion protein 1, all other identified proteins were independent from known EC risk variants identified in EC GWAS. The respective odds ratios (95% confidence intervals) per one standard deviation increase in genetically predicted circulating protein concentrations were 1.21 (1.13, 1.30) for DNA repair protein RAD51 homolog 4, 1.27 (1.14, 1.42) for desmoglein-2, 1.14 (1.07, 1.22) for MHC class I polypeptide-related sequence B, 1.05 (1.02, 1.08) for histo-blood group ABO system transferase, 0.77 (0.68, 0.89) for intestinal-type alkaline phosphatase, 0.82 (0.74, 0.91) for carbohydrate sulfotransferase 15, 1.07 (1.03, 1.11) for D-glucuronyl C5-epimerase, and 1.07 (1.03, 1.10) for CD209 antigen. In conclusion, we identified nine potential EC-associated proteins. If validated by additional studies, our findings may contribute to understanding the pathogenesis of endometrial tumor development and identifying women at high risk of EC along with other EC risk factors and biomarkers.

## 1. Introduction

Endometrial cancer (EC) is a leading gynecological malignancy in developed countries [1]. It is also one of the few cancer types with a rapidly increasing incidence and mortality as the rate of obesity continues to grow worldwide [2]. Hence, it is reasonable to predict that EC will become an important public health challenge in the coming years. There is an urgent need to reduce the disease burden by enhancing the understanding of the EC etiology and distinguishing high-risk women from the general population.

Traditionally, EC is classified into two main histological subtypes. Endometrioid adenocarcinomas represent more than 70% of cases. Tumors of this type are usually low-grade and diagnosed at an early stage. In contrast, the less common non-endometrioid tumors are typically more aggressive and often diagnosed at an advanced stage. Although the prognosis of EC remains generally good, it worsens dramatically when diagnosed at an advanced stage, with a median survival time of less than 12 months [1,3]. To improve the treatment efficacy and survival outcomes of this disease, it is critical to detect EC at the earliest possible stage. The discovery of potential non-invasive biomarkers would thus be especially important for identifying women with a high risk of EC [4,5]. Although many protein candidates in blood or vagina samples have been reported as possible biomarkers, most of these studies have only assessed a limited number of candidates or yielded inconsistent results [4,6,7]. For instance, several studies found associations of serum level of cancer antigen 15-3 and serum amyloid A with an increased risk of EC, while others showed inverse or null associations [8,9,10,11,12,13,14]. These studies using a conventional epidemiological design are potentially subject to selection biases, residual confounding, or reverse causality. Furthermore, they were often limited by a small sample size and a limited number of protein candidates available for evaluation.

To identify novel protein biomarkers for EC, here, we used protein quantitative trait loci (pQTL) from a recently published genome-wide association study (GWAS) as genetic instruments to investigate the associations between genetically predicted protein concentrations and EC risk. Due to the independent assortment of alleles transmitted from parents to offspring during gamete formation, such a design can potentially address several limitations of conventional epidemiological studies [15]. We leveraged comprehensive data of 12,906 EC cases and 108,979 controls of European ancestry generated in the Endometrial Cancer Association Consortium (ECAC), the Epidemiology of Endometrial Cancer Consortium (E2C2), and the UK Biobank.

## 2. Methods

After an extensive literature search and rigorous evaluation, we identified a comprehensive study analyzing associations between genetic variants and blood-based protein concentrations and used pQTLs identified in this study as the instruments for our analyses [16]. By analyzing data on 3562 healthy European descendants with adjustment for age, sex, duration between blood draw and processing, and the first three principal components (PCs), this study identified 764 genomic loci that were associated (*p* < 1.5 × 10^−11^) with expression levels of 1478 proteins, involving a total of 1927 associations [16]. Instrumental variables were created based on these pQTLs to examine the associations between genetically predicted protein concentrations and EC risk. When there were more than one variant located at the same chromosome associated with a single protein, we only retained single nucleotide polymorphisms (SNPs) that were independent of each other, as defined by R^2^ < 0.1 (based on 1000 Genomes Project Phase 3 version 5 data focusing on European populations).

To understand the associations between genetically predicted protein levels and EC risk, we used summary statistics from the largest GWAS meta-analysis previously conducted to evaluate the association between genetic variants and the risk of developing EC [17]. In brief, 12,906 EC cases and 108 979 controls of European descent from 17 studies as part of the ECAC, E2C2, and the UK Biobank were included. Stratified analyses were also performed by histologic subtype including endometrioid (*n* = 8758) and non-endometrioid carcinomas (*n* = 1230). These participants were genotyped via various platforms. Among them, 4710 EC cases were genotyped using the OncoArray chip, and were country-matched to 19,438 controls who were genotyped in the same way from the Breast Cancer Association Consortium [17]. The 2381 cases and 13,675 controls from the iCOGs studies were genotyped using the Illumina Infinium iSelect array. Regarding participants from E2C2, the 2271 cases and 2219 controls in the United States were genotyped using the Illumina Human OmniExpress array, and 424 cases and 558 controls from Poland were genotyped using the Illumina Human 660W array. Data from the UK biobank, including 636 EC cases and 62 853 controls, were genotyped using the Affymetrix UK BiLEVE Axiom array and Affymetrix UK Biobank Axiom array. The 288 cases and 1440 controls identified from the Women’s Health Initiative were genotyped using five different arrays (Illumina Human Omni1-Quad v1-0 B; Illumina 610; Human OmniExpressExome-8v1-1-A; Axiom Genome-Wide Human CEU; Human OmniExpress-8v1_B). Information on genotyping and imputation methods for the samples from other published GWAS studies can be found in the original GWAS paper [17]. Risk estimates for the SNP-EC associations estimated in each study with adjustment for PCs were meta-analyzed by inverse variance weighted (IVW) fixed-effects methods [18]. All participating studies were approved by their appropriate ethics committees with written and informed consent from all participants.

Based on the summary estimates from the pQTL study and the EC GWAS meta-analysis mentioned above, we assessed the associations between genetically predicted circulating protein concentrations and EC risk by using the IVW method [19,20]. Briefly, the estimated beta coefficient and corresponding standard error (SE) of the association between each protein and EC risk were calculated using the formula of $\sum_{i}\beta_{i,GX}*\beta_{i,GY}*\sigma_{i,GY}^{-2}/\left(\sum_{i}\beta_{i,GX}^{2}*\sigma_{i,GY}^{-2}\right)$ and $1/{\left(\sum_{i}\beta_{i,GX}^{2}*\sigma_{i,GY}^{-2}\right)}^{0.5}$, respectively, where *β_i,GX_* represents the beta coefficient for the association between each SNP and protein level adopted from the pQTL study; *β_i,GY_* and *σ_i,GY_* represent the estimated beta coefficient and SE of the association between each individual SNP and EC risk in the latest GWAS [20]. Odds ratios (ORs) and confidence intervals (CIs) were further calculated by exponentiating the beta coefficients. Analyses were also carried out separately for endometrioid and non-endometrioid carcinomas, considering the possible etiological heterogeneity. Statistical significance was determined by a Benjamini–Hochberg false discovery rate (FDR) of <0.05. For proteins showing an association, conditional and joint multiple-SNP analysis (COJO) conditional analysis was used to examine the robustness of the identified association after conditioning on known GWAS-identified EC risk variants. The ingenuity pathway analysis (IPA) was conducted to visualize the canonical pathways, relevant diseases, biological functions, and networks enriched by genes encoding the proteins associated with EC risk in our pQTL analysis [21].

## 3. Results

We assessed the associations between genetically predicted circulating levels of 1434 proteins and EC risk using pQTLs as instrument variables. Among the examined proteins, nine showed associations with EC risk at a FDR of <0.05, and five satisfied Bonferroni criterion (0.05/1434). Positive associations were observed for six of the identified proteins, including DNA repair protein RAD51 homolog 4 (RA51D), desmoglein-2, MHC class I polypeptide-related sequence B (MICB), histo-blood group ABO system transferase (BGAT), D-glucuronyl C5-epimerase (GLCE) and CD209 antigen (DC-SIGN), with ORs ranging from 1.05 to 1.27 (Table 1). We observed negative associations for three proteins: vascular cell adhesion protein 1 (VCAM-1), intestinal-type alkaline phosphatase, and carbohydrate sulfotransferase 15 (ST4S6), with ORs ranging from 0.60 to 0.82 (Table 1). The instruments used for VCAM-1, MICB, BGAT, intestinal-type alkaline phosphatase, and ST4S6 only had one SNP; whereas two SNPs were used as the instrument to predict the circulating level of RA51D, desmoglein-2, GLCE, and DC-SIGN. Except for VCAM-1 in which the instrument variant (rs3184504) was previously reported as an EC risk SNP, the observed associations for all other identified proteins were independent from known EC risk SNPs from the published GWAS [17]. COJO conditional analysis showed that associations between those eight predicted proteins and EC risk generally remained the same after conditioning on known EC risk variants identified in previous GWAS (Table 1).

Subgroup analyses by histologic subtype of EC suggested that most of the identified associations in the combined analysis remained the same for either endometrioid or non-endometrioid histotype, although many of the associations failed to reach multi-testing-adjusted statistical significance due to reduced sample sizes. Of the nine proteins identified in our main analysis, the uncorrected *p*-value ranged from 3.16 × 10^−2^ (DC-SIGN) to 5.78 × 10^−9^ (VCAM-1) for their associations with endometrioid EC risk. Regarding the rare and aggressive non-endometrioid EC, the uncorrected *p*-values were less than 0.02 for VCAM-1, BGAT, alkaline phosphatase intestine, ST4S6 and DC-SIGN. The directions of the associations between these five proteins and EC risk were consistent across the combined and subgroup analyses (Table 2).

Pathway analysis by IPA suggested enrichment in several oncogenic pathways for the genes encoding the proteins identified in our study. The top canonical pathways involved those of heparan sulfate biosynthesis at both late (*p*-value = 3.41 × 10^−4^) and early stages (*p*-value = 4.12  ×  10^−4^) as well as crosstalk between dendritic cells and natural killer cells (*p*-value = 5.36  ×  10^−4^) (Table 3).

## 4. Discussion

To our knowledge, this is the first study to comprehensively examine the associations between genetically predicted circulating protein concentrations and EC risk among European descendants using data from the largest GWAS conducted by international consortia. Among the 1434 proteins investigated, we identified nine EC-associated proteins after FDR correction, including eight independent from previously identified EC risk variants. Similar findings were observed for endometrioid EC alone. Five of the proteins also showed suggestive associations with the risk of non-endometrioid EC, a much more lethal subtype [22]. If validated in additional studies, our findings add new knowledge to the etiology of endometrial tumorigenesis, and yield a list of candidate protein biomarkers for EC risk assessment.

Previous studies have reported a number of blood-based EC protein biomarker candidates such as DKK-1, DJ-1, HE4, CA125, GDF-15, SPAG9, YKL-40, IL-31 and IL-33 [4,6,23]. Unfortunately, there was no corresponding pQTL available as instruments for HE4, CA125, SPAG9 and IL-33. For the other biomarker candidates, we did not observe associations in the current study: DKK-1 (OR = 0.96, 95% CI: 0.87–1.07; *p*-value = 0.44), DJ-1 (OR = 0.94, 95% CI: 0.87–1.03; *p*-value = 0.17), GDF-15 (OR = 0.98, 95% CI: 0.93–1.05; *p*-value = 0.59), YKL-40 (OR = 1.00, 95% CI: 0.97–1.03; *p*-value = 0.99) and IL-31 (OR = 0.95, 95% CI: 0.89–1.02; *p*-value = 0.17). Explanations for our failure to replicate the associations for DKK-1, DJ-1, GDF-15, YKL-40 and IL-31 might include either the weakness of their corresponding pQTLs used as instruments in our study or false-positive findings due to common biases in conventional observational studies. For example, obesity, the most important risk factor for EC, is also suggested to be associated with measured levels of GDF-15 and YKL-40 [24,25,26]. Therefore, the reported associations of these proteins with EC risk in previous observational studies could be confounded by obesity due to imperfect adjustment in their analyses [27].

The instrument SNP for VCAM-1 protein, rs3184504, was previously identified as an EC-risk variant and had a trans effect on the protein expression. *SH2B3* is a likely regulatory target at this risk region, as evidenced from our previous work using chromatin capture methods in endometrial cell lines [28]. *SH2B3* is known to downregulate VCAM-1, which could perhaps be one of the potential mechanisms by which this association is occurring [29]. However, given the pleiotropic nature of *SH2B3*, we cannot preclude other possible mechanisms for the observed association (e.g., immune and inflammatory signaling pathways) [30]. In the current study, we identified eight EC-associated proteins that are independent of previously identified EC risk variants. Some of them have been reported to play an essential role in endometrial tumorigenesis. Polymorphisms of the gene encoding RA51D have been identified to be associated with EC risk in population-based studies [31,32]. The RA51D is a core protein involved in the homologous recombination and repair of double strand breaks in DNA molecules, which is viewed as the most detrimental DNA damage that can trigger EC development [33]. With regard to protein BGAT, an earlier study found that blood group-related antigens were expressed differently between normal and neoplastic endometria [34]. However, observational studies investigating the association between different ABO blood types and EC risk yielded inconsistent findings in different populations [35,36,37]. Not much is known about the remaining proteins we identified. Future studies are warranted to further elucidate the involvement of the proteins we found in endometrial carcinogenesis.

The strengths of our study include the use of the largest GWAS in maximizing the statistical power of our study. Additionally, our study design of using genetic instruments could substantially reduce common biases embedded in traditional observational studies. Nevertheless, we also have to acknowledge several limitations in the current study. First, our findings may be influenced by potential pleiotropic effects. For instance, rs550057, which served as the instrument SNP for both alkaline phosphatase intestine and ST4S6, is also in some linkage disequilibrium with several instrument SNPs of other identified proteins. Thus, whether the identified associations in our study were attributable to correlations between protein concentrations needs further investigation. Similarly, rs45446698, which was one of the instrument variants for protein RA51D, has been linked to urinary metabolite levels in chronic kidney disease, offspring birth weight, heel bone mineral density and height [38,39,40,41]. Nevertheless, none of these traits have shown a strong independent relationship with EC risk. Second, given that the observed associations for the identified proteins were only based on the genetically regulated components in our study, the utility of their measured circulating protein concentrations for EC risk assessment needs to be further verified and then validated. Third, relying on previously identified pQTLs as instrument variables, we were only able to evaluate protein candidates with at least one existing pQTL identified. Due to this limitation, we were not able to compare our findings to some of the previously reported EC protein biomarker candidates. We anticipate that additional protein biomarkers will be uncovered with the identification of new pQTLs in future studies, as well as in a study of using more thorough methods to predict protein concentrations using a combination of multiple genetic variants, which is expected to further increase study power. Moreover, we failed to replicate the associations for several previously reported possible EC protein biomarkers, which might be explained by either the weakness of the corresponding pQTLs used as instruments in our study or distinct study designs between our study and the previous studies. In addition, given that the associations identified in our study have moderate effect sizes and we were unable to provide detailed functional validation at this moment, our findings may not have immediate potential for translating into clinical use based on the current results. On the other hand, our findings should provide additional insights into the etiology of endometrial cancer. The identified associations provide a basis for future investigation of directly measured levels of the nine proteins in risk assessment of endometrial cancer. It is possible that although the individual protein’s effect size is modest, when combined together, a much larger effect can be achieved. Finally, our subgroup analyses by histologic subtype are limited by small numbers of cases, especially for non-endometrioid carcinoma, and we were not able to assess the associations for a more detailed classification of EC. Future studies with improved statistical power are needed to better investigate the associations for this more aggressive and specific type of EC.

## 5. Conclusions

In summary, in this large study using genetic variants as instruments, we identified nine protein biomarkers with their genetically predicted circulating concentrations associated with EC risk. Our study provided a list of EC-associated protein biomarkers, which, if validated in additional studies, will not only contribute to the understanding of endometrial tumorigenesis but also facilitate risk assessment of EC combined with other findings.

## Figures and Tables

**Table 1:** Associations between genetically predicted protein concentrations and endometrial cancer risk.

Protein	Protein Full Name	Protein-Encoding Gene	Region for Protein Encoding Gene	Instrument Variants	Type of pQTL	OR ^a^	Lower Bound 95% CI ^a^	Upper Bound 95% CI ^a^	*p*-Value	FDR *p*-Value ^b^	*p*-Value after Conditioning on Risk Variants ^c^
VCAM-1	Vascular cell adhesion protein 1	*VCAM1*	1p21.2	rs3184504	*trans*	0.60	0.51	0.70	1.14 × 10^−10^	1.53 × 10^−7^	NA
RA51D	DNA repair protein RAD51 homolog 4	*RAD51D*	17q12	rs6838228; rs45446698	*trans*; *trans*	1.21	1.13	1.30	2.46 × 10^−8^	1.66 × 10^−5^	2.46 × 10^−8^
Desmoglein-2	Desmoglein-2	*DSG2*	18q12.1	rs687621; rs2704050	*trans*; *cis*	1.27	1.14	1.42	2.18 × 10^−5^	0.01	2.18 × 10^−5^
MICB	MHC class I polypeptide-related sequence B	*MICB*	6p21.33	rs3134900	*cis*	1.14	1.07	1.22	3.30 × 10^−5^	0.01	2.41 × 10^−5^
BGAT	Histo-blood group ABO system transferase	*ABO*	9q34.2	rs505922	*cis*	1.05	1.02	1.08	8.50 × 10^−5^	0.02	8.51 × 10^−5^
Alkaline phosphatase intestine	Intestinal-type alkaline phosphatase	*ALPI*	2q37.1	rs550057	*trans*	0.77	0.68	0.89	1.84 × 10^−4^	3.54 × 10^−2^	1.84 × 10^−4^
ST4S6	Carbohydrate sulfotransferase 15	*CHST15*	10q26.13	rs550057	*trans*	0.82	0.74	0.91	1.84 × 10^−4^	3.54 × 10^−2^	1.84 × 10^−4^
GLCE	D-glucuronyl C5-epimerase	*GLCE*	15q23	rs2519093; rs11854180	*trans;* *cis*	1.07	1.03	1.11	2.14 × 10^−4^	3.61 × 10^−2^	2.14 × 10^−4^
DC-SIGN	CD209 antigen	*CD209*	19p13.2	rs505922; rs145827860	*trans*; *cis*	1.07	1.03	1.10	3.04 × 10^−4^	4.56 × 10^−2^	3.04 × 10^−4^

^a^ OR (odds ratio) and CI (confidence interval) per one standard deviation increase in genetically predicted protein levels. ^b^ FDR *p*-value: false discovery rate (FDR) adjusted *p*-value; associations with an FDR *p* < 0.05 considered statistically significant. ^c^ Adjusted GWAS-identified EC risk variants include: rs113998067, rs148261157, rs1679014, rs10835920, rs9668337, rs3184504, rs10850382, rs1129506, rs882380, rs1740828, rs2747716, rs35286446, rs4733613, rs139584729, rs7981863, rs2498796, rs937213, rs17601876, rs11263761.

**Table 2:** Associations between genetically predicted protein concentrations and endometrial cancer risk by subtypes of endometrial cancer (endometrioid and non-endometrioid).

Protein	Endometrioid (8758 Cases/46 126 Controls)				Non-Endometrioid (1230 Cases/35 447 Controls)			
	OR ^a^	Lower Bound 95% CI ^a^	Upper Bound 95% CI ^a^	*p*-Value	OR ^a^	Lower Bound 95% CI ^a^	Upper Bound 95% CI ^a^	*p*-Value
VCAM-1	0.58	0.48	0.69	5.78 × 10^−9^	0.58	0.37	0.91	1.89 × 10^−2^
RA51D	1.19	1.10	1.30	1.87 × 10^−5^	NA ^b^	NA ^b^	NA ^b^	NA ^b^
Desmoglein-2	1.21	1.06	1.38	4.09 × 10^−3^	1.35	0.98	1.85	6.84 × 10^−2^
MICB	1.15	1.07	1.24	2.96 × 10^−4^	1.04	0.86	1.24	0.70
BGAT	1.04	1.01	1.07	1.11 × 10^−2^	1.09	1.01	1.16	1.82 × 10^−2^
Alkaline phosphatase intestine	0.84	0.71	0.98	2.46 × 10^−2^	0.61	0.42	0.90	1.14 × 10^−2^
ST4S6	0.87	0.77	0.98	2.46 × 10^−2^	0.68	0.51	0.92	1.14 × 10^−2^
GLCE	1.07	1.02	1.12	2.14 × 10^−3^	0.99	0.89	1.09	0.78
DC-SIGN	1.05	1.00	1.09	3.16 × 10^−2^	1.13	1.02	1.25	1.68 × 10^−2^

^a^ OR (odds ratio) and CI (confidence interval) per one standard deviation increase in genetically predicted protein levels. ^b^ NAs were due to the unavailability of the GWAS result for the instrument SNP rs45446698.

**Table 3:** Canonical pathways, diseases, bio functions, and networks associated with the genes encoding identified endometrial cancer risk-associated proteins ^a^.

Top Canonical Pathways			Top Diseases and Disorders	Molecular and Cellular Functions	Top Networks
	*p*-Value	Involved Molecules			
Heparan Sulfate Biosynthesis (Late Stages);	3.41 × 10^−4^	*CHST15, GLCE*	Infectious diseases; cancer; cardiovascular disease; connective tissue disorders; hereditary disorder.	Cell-to-cell signaling and interaction; cellular movement; cellular assembly and organization; carbohydrate metabolism; cell morphology.	Cell-to-cell signaling and interaction, cancer, connective tissue disorders.
Heparan Sulfate Biosynthesis;	4.12 × 10^−4^	*CHST15, GLCE*			
Crosstalk between Dendritic Cells and Natural Killer Cells;	5.36 × 10^−4^	*CD209, MICB*			
Dermatan Sulfate Biosynthesis (Late Stages);	1.81 × 10^−2^	*CHST15*			
Chondroitin Sulfate Biosynthesis (Late Stages);	1.88 × 10^−2^	*CHST15*			

^a^ Genes involved in the pathway analysis include VCAM1, RAD51D, DSG2, MICB, ABO, ALPI, CHST15, GLCE, and CD209.

## Data Availability

The data that support the findings of this study are available from the corresponding author upon reasonable request.
